# Neurocognitive Impairment and HIV Treatment Engagement in Men Who Have Sex With Men Living With HIV Who Report Chronic Pain and Substance Use

**DOI:** 10.1155/arat/3404193

**Published:** 2025-01-28

**Authors:** Matthew C. Sullivan, Megan R. Wirtz, Samantha M. McKetchnie, Lauren R. Gulbicki, S. Wade Taylor, Jonathan D. Jampel, Nikhil Banerjee, Conall O'Cleirigh

**Affiliations:** ^1^Department of Psychiatry, Massachusetts General Hospital, Boston, Massachusetts, USA; ^2^Department of Psychology, The Graduate Center, City University of New York, New York, New York, USA; ^3^Behavioral Sciences Research Program, The Fenway Institute, Fenway Health, Boston, Massachusetts, USA; ^4^Department of Neurology, Vanderbilt University Medical Center, Nashville, Tennessee, USA

**Keywords:** adherence, cognitive processing, HIV, older person, pain

## Abstract

This study explored relationships between neurocognitive impairment (NCI), engagement in HIV care, and functional disability among sexual minority men aged 50 years or older living with HIV, chronic pain, and recent substance use. Sixty-three participants completed cross-sectional assessments including a neurocognitive screening measure, self-reported HIV medication adherence, past-year attendance at HIV-care appointments, and indices of pain and functional impairment. Mean participant age was 57.2 years; most identified as White (55%), followed by Black/African American (42%). On average, participants reported moderate pain; 66.7% met DSM-5 criteria for a substance use disorder. Average Montreal Cognitive Assessment (MoCA) performance reflected mild NCI. Regression analyses indicated an association between poorer MoCA performance and past-year missed HIV-care appointments (*B* = −0.09, *t* (59) = −3.10, *p* = 0.004). Self-reported cognitive impairment was associated with more missed HIV-care visits (*B* = 0.20, *t* (59) = 4.82, *p* < 0.001) and greater functional disability, whereas poorer semantic fluency was associated with fewer missed HIV-care visits (*B* = −0.49, *t* (59) = −3.99, *p* < 0.001). Increased brief neuropsychological assessment and linkage to tailored interventions for HIV-care engagement and substance use mitigation are warranted to support PLWH with NCI in clinical care.

## 1. Introduction

People living with HIV (PLWH) are living longer, with the majority of the United States PLWH population aged 50 years or older [1]. However, with longevity come chronic health conditions that adversely impact quality of life, such as HIV-associated and other neurocognitive impairment (NCI) [2]. Among this aging population of PLWH, HIV-associated neurocognitive disorders and other manifestations of NCI are common comorbidities. People over 50 account for nearly 50% of PLWH with NCI, and PLWH are seven times more likely to experience NCI than HIV-uninfected people [3–5]. In addition, while 13% of the general population experiences cognitive decline after the age of 60, a disproportionate 28%–88% of PLWH experience the same decline [6–8].

Substance use (SU) may contribute to this discrepancy in NCI rates among PLWH [9]. Substance use disorders (SUDs) are prevalent among PLWH, particularly among men who have sex with men (MSM), who make up 69% of new HIV cases in the United States [10, 11]. In MSM, substances may be used as coping mechanisms to address a range of stressors, including stigma, adverse childhood experiences, and chronic pain [12–15]. Nearly 50% of older MSM living with HIV reported nonprescription drug use in the past month, and this SU may increase with age [16, 17]. SU directly contributes to poorer cognitive performance but may also undermine the protective benefits of ART due to its adverse impact on healthcare visit attendance and medication adherence [18, 19].

NCI and SU are both highly comorbid with chronic pain in PLWH, and together, these factors may hinder HIV-care engagement [20, 21]. NCI has been shown to partially mediate the relationship between SU and suboptimal ART adherence among PLWH [22], while both SU and NCI are directly related to suboptimal ART adherence [23, 24]. Among older MSM living with HIV, higher pain scores are associated with more missed HIV appointments, likely attributable to both chronic pain and SU [25]. Thus, the links between NCI and suboptimal engagement in HIV-related care are likely multifactorial among older MSM who use substances and live with HIV and chronic pain.

Research has demonstrated that NCI may also adversely impact health-related quality of life (HRQoL) among PLWH, including older MSM (e.g., associations with unemployed status, overall HRQoL, and poor subjective physical health quality) [26, 27]. Thus, older MSM living with HIV—a group disproportionately impacted by harmful social conditions such as stigma and adverse childhood experiences—may in turn develop overlapping, or syndemic, chronic disease disparities exacerbated by the interplay between NCI, chronic pain, and SU that in turn contribute to suboptimal HIV-care engagement [28, 29].

Given these intersecting vulnerabilities, we investigated the associations between NCI and engagement in HIV care and functional disability in MSM aged 50 years and older living with HIV, chronic pain, and SU. We aimed to (a) characterize the neurocognitive functioning in our sample; (b) determine whether measures of NCI are associated with poorer HIV-care engagement; and (c) determine whether NCI is associated with indices of poorer health and functional impairment.

## 2. Methods

### 2.1. Setting and Procedures

Data collected for this secondary analysis are part of a cross-sectional study aimed at characterizing the relationships between chronic pain, SU, and engagement in HIV care among older MSM. Study activities were conducted at Fenway Health in Boston, MA. Participants were phone screened for eligibility and invited for a study visit, consisting of informed consent, self-report surveys, clinician-administered assessments, and oral toxicology for opioids. Participants were asked to sign a medical record release to collect information on HIV disease markers (viral load and CD4 count) and prescribed ART and pain treatments. Participants were compensated $50 for their time and were provided community referrals to SU treatment, pain clinics, and support services for PLWH. All study procedures were approved by the Fenway Health Institutional Review Board (#588318-22).

### 2.2. Participants

Recruitment consisted of community outreach (AIDS service organizations, bars and clubs catering to lesbian, gay, bisexual, transgender, queer or questioning [LGBTQ+] individuals, and cruising areas) and online advertising (Craiglist and MSM-focused dating sites). Participants were also recruited from the Fenway Health Medical Department. Participants were eligible if they (1) were MSM; (2) were at least 50 years old; (3) reported chronic pain that had endured for 3 months or longer; (4) were living with HIV; and (5) reported use of illicit substances or nonmedical use of pain medications in the past 6 months. Exclusion criteria included individuals who were younger than 50 years of age, did not self-identify as MSM, reported being HIV-uninfected or having unknown HIV status, or were unable to give informed consent.

### 2.3. Assessment Measures

#### 2.3.1. Sociodemographics

Participants reported their age, sexual orientation, race, ethnicity, annual personal income, and education level.

#### 2.3.2. HIV Clinical Characteristics

Participant medical records were reviewed for most recent HIV viral load value and CD4 counts. Medical record data were gathered within a period of 1 year before and after the study visit, selecting data from the nearest time point to the study visit. In addition, participants self-reported years since HIV diagnosis.

#### 2.3.3. NCI Screening Measures

Neurocognitive functioning measures were selected to assess a range of cognitive domains hypothesized to be relevant to HIV-associated NCI and for their brevity.

To measure participants' neurocognitive functioning, the Montreal Cognitive Assessment (MoCA) Version 7.1 [30] was administered by a trained social work clinician. The MoCA is a brief screening measure assessing global cognitive status. The measure is comprised of brief tasks assessing visuospatial and executive functioning, recognition memory, attention, language recall, abstraction, delayed recall, and orientation. Scoring is divided into eight subscales summing to a total score of 30 points; a cutoff of 25 points or lower is most often used for detecting mild cognitive impairment [30]. Permission was obtained by the study team for research use of this measure.

To assess semantic fluency, participants completed the verbal fluency task (VFT), which asks participants to name as many animals as they can in 60 seconds. Number of animals named is interpreted in relation to age- and education-referenced norms as a scaled z-score [31].

The Trail-Making Test-Form B (TMT-B) measures timed cognitive set-shifting and is sensitive to frontal lobe dysfunction. The test presents participants with circled numbers and letters and asks participants to draw lines to connect circles, alternating between letter–number sets in ascending order. Time to completion is scaled in relation to age- and education-referenced normative data [32]. The longer the time to complete the task, the greater the impairment [33].

Patients also self-reported symptoms of cognitive impairment using the Patient Assessment of Own Functioning Inventory (PAOFI) [34]. The PAOFI is a 33-item measure that assesses subjective cognitive functioning, comprising five domains: language and communication, higher level cognition, memory, sensory–perceptual impairment, and use of hands. The PAOFI is calculated with a total score of 33 self-report items (each scored 0-5 with a possible global score range of 0–165). Higher scores indicated greater perceived NCI).

#### 2.3.4. HIV-Care Engagement Measures

We used an HIV-specific Resource Utilization Questionnaire (RUQ) to assess care engagement. The RUQ is an investigator-developed measure used in previous published studies by this group that includes questions related to year of HIV diagnosis, year participant received an HIV case manager, and number of health visits per year, as well as missed medical appointments per year [25]. To assess engagement in HIV-related care, we examined the number of missed visits to one's HIV-care provider in the past year.

#### 2.3.5. ART Adherence

The AIDS Clinical Trial Group (ACTG) self-report questionnaire was used to measure HIV medication adherence. This measure asks participants to report the number of doses of each of their respective antiretroviral medications they missed in the past two weeks. For participants taking multiple antiretrovirals, we calculated the mean miss rate across medications. We then inverted this miss rate to create a percentile score of 0%–100% adherence [35].

#### 2.3.6. Measure of Pain

Pain was measured via the Brief Pain Inventory [36], which has participants rate experiences of pain on a 0–10 scale (0 = *no pain*, 10 = *worst pain imaginable*). For this analysis, a composite score was created using mean values for current pain, average pain in the past week, and highest and lowest pain in the past week [37].

#### 2.3.7. Functional Impairment

Participants were asked to complete the WHO Disability Assessment Schedule 2.0 (WHODAS) [38]. The 12-item WHODAS examines several domains of functional disability on a 0–5 scale (indicating no disability to extreme disability) to yield a total functional disability score (higher scores indicating greater functional disability).

#### 2.3.8. SU Characteristics

SU was measured by clinician assessment via the MINI International Neuropsychiatric Interview [39]. A dichotomous variable (presence or absence of current disorder) was created for each category of SUD—alcohol, opioids, stimulants, and cannabis. In addition, a composite variable was created to indicate the presence of *any* current SUD.

### 2.4. Data Analysis

Descriptive statistics were calculated to summarize the demographic and health characteristics of the sample. Our primary analyses examined the association between MoCA total score and engagement in HIV care (i.e., ART adherence and past-year missed HIV-care appointments) using multivariate regression analyses, in which independent variables and controls were entered in a single step. Variables were assessed for normality. Where the distribution of variables departed for normality, transformations were applied in accordance with the distribution of the data. Linear regression with a reflected natural log transformation was applied in the case of ART adherence, to account for the left-skewed distribution of adherence data [40]. Generalized linear regression using a Poisson distribution was applied for analysis of missed HIV-care appointments, to best account for the distribution of this count data [41]. Binary logistic regression was used to examine neurocognitive functioning in relation to HIV viral suppression status (undetectable vs. detectable viral load, dichotomized at 40 copies/mL). We also performed secondary analyses using linear regression to examine the associations between neurocognitive functioning and health and quality of life outcomes, including functional impairment and pain. All analyses of neurocognitive function measures controlled for CD4 count and HIV viral suppression status, variables that were selected a priori. Relationships between objective and subjective measures of neurocognitive performance were assessed using Pearson's correlation coefficients (*r*). As a significant proportion of HIV health-related data were missing in the current sample (16% of viral suppression data were missing and 22% chart-abstracted CD4 count data), we performed multiple imputation to account for missingness among these control variables using the *mice* package in *R* [42]. As these data appeared to be missing completely at random (MCAR), multiple imputation was deemed appropriate [43]. All statistical tests defined significance as*p* ≤ 0.05. Descriptive analyses were performed using IBM SPSS Version 27. All other analyses were performed in *R* [42].

## 3. Results

### 3.1. Sample Characteristics

The study sample included 63 MSM living with HIV who reported chronic pain and recent SU. Sample demographic and health characteristics are summarized in Table 1. The average age of participants was 57.2 years (SD: 5.6), and the majority identified their race as White (55%), followed by Black or African American (42%), American Indian (5%), Asian (2%), and Native Hawaiian/Pacific Islander (2%); 7% identified their ethnicity as Hispanic/Latinx. The sample varied in terms of educational attainment (54% had less than a college degree) and most participants reported low income (74% less than $20,000). A minority of the sample demonstrated poor HIV disease control (13% had a detectable HIV viral load; 8% had a CD4 count < 200 cells/mm^3^). On average, participants reported moderate levels of pain (BPI composite mean score = 5.2; SD: 1.6). Two-thirds (66.7%) met clinician-assessed DSM-5 criteria for an SUD. On average, the sample demonstrated neurocognitive functioning in a range indicative of mild NCI (mean MoCA score = 23.6; SD: 3.9; 66.7% < 26).

### 3.2. NCI, ART Adherence, and Missed HIV-Care Visits

Neurocognitive function screening measures (MoCA, TMT-B, and a measure of semantic fluency) were examined in relation to measures of engagement in HIV medical care via linear and Poisson regression (Table 2). No neurocognitive screening measures were significantly associated with self-reported adherence to ART medications. Greater NCI, measured by the MoCA, was associated with more missed HIV-care appointments in the past year (*B* = −0.10, *t* (59) = −3.10, *p*=0.004). Poorer semantic fluency performance was associated with fewer missed HIV-care appointments in the past year (*B* = −0.15, *t* (59) = −3.99, *p* < 0.001). The TMT-B was not associated with either ART adherence or missed HIV-care appointments. Higher PAOFI scores (indicative of greater self-reported cognitive impairment) were associated with more missed HIV-care appointments in the past year (*B* = 0.15, *t* (59) = 4.66, *p* < 0.001). No neurocognitive screening measure was associated with HIV viral suppression status or CD4 count.

### 3.3. NCI and Health-Related Quality of Life

Linear and Poisson regression analyses showed associations between neurocognitive screening measures and indices of pain and functional disability (Table 3). Semantic fluency showed a negative association with functional impairment (WHODAS; *B* = −0.32, *t* (59) = −2.60, *p*=0.012). TMT-B, a measure of cognitive flexibility, was negatively associated with pain, such that better performance was associated with greater pain severity (BPI composite score; *B* = −0.27, *t* (59) = −2.18, *p* = 0.033). Higher PAOFI score (self-reported cognitive impairment) was associated with greater functional impairment (WHODAS; *B* = 0.20, *t* (59) = 4.83, *p* < 0.001).

### 3.4. Neurocognitive Screening Relative to Self-Report

Despite associations with several subjective indices of treatment engagement and functioning, PAOFI scores were not significantly correlated with two of three objective measures of cognitive performance (MoCA and TMT-B). PAOFI scores did show a significant negative correlation with semantic fluency (*r* = −0.35; *p* < 0.005). The PAOFI did not show a statistically significant relationship with performance on either the MoCA (*r* = −0.12; *p* = 0.35, n.s.) or the TMT-B (*r* = −0.14; *p* = 0.30, n.s.).

### 3.5. NCI in Comparable Samples of PLWH

Table 4 compares measures of cognitive functioning in the current sample with those in prior studies of PLWH (these samples ranged from 75% to 96% male-identified; mean ages 41–58 years). Mean MoCA scores in the current sample suggested significantly greater NCI than on average in studies by Fazeli, Koenig, and Janssen et al. (*p*'s < 0.05), yet scores did not differ significantly from those in Milanini et al. (*p* = 0.55, *n.s*.) [6, 44–46]. Self-reported cognitive difficulties in the current sample were compared with a 1999 study by Rourke et al. that also used the PAOFI [47]. PAOFI scores in the current sample were comparable to clinically asymptomatic PLWH in the Rourke et al. study (*p* = 0.11, *n.s*.), and scores were lower than cognitive complaints among those with clinically symptomatic HIV-related illness (i.e., those with CDC Classifications B and C).

## 4. Discussion

This study examined cognitive functioning in relation to clinical and quality of life outcomes in a sample of older MSM living with HIV who report chronic pain and SU. Two health-related domains were assessed: engagement in HIV-related care and functional disability. We found that poorer cognitive functioning, as assessed by MoCA and self-report, was associated with suboptimal engagement in HIV-related care; in particular, poorer MoCA performance was associated with more missed HIV-care appointments. Greater self-reported cognitive difficulties (via PAOFI) were also associated with more missed HIV-care appointments as well as greater functional impairment. Poorer neurocognitive functioning was also associated with greater functional impairment. Surprisingly, greater cognitive flexibility (as measured by TMT-B) was associated with greater overall pain symptoms.

To examine the validity of the NCI measurement in the current study, we compared measurements for our sample to previous study samples of PLWH [6, 44–46]. In general, participants in the current study showed comparable neurocognitive function to PLWH in other samples. While PLWH in the current study performed significantly poorer on the MoCA than PLWH in three of four comparative study samples, these studies tended to be more restrictive in terms of inclusion criteria, for instance restricting on the basis of current SU, thereby reflecting PLWH who were less likely to have sustained SU-related cognitive deficits [6, 44–46]. To characterize self-reported cognitive complaints in the current sample, PAOFI scores were compared with those from Rourke et al. [47]. Although the Rourke sample differed in terms of contemporaneously available HIV pharmacotherapies and services, participants in the current sample showed statistically significant differences only from those PLWH with symptomatic HIV-related illness. Thus, our NCI findings appear to reflect a representative picture of NCI among PLWH. The lower mean MoCA performance in our sample may have been attributable to the older age group relative to other studies examined.

As hypothesized, we found that impaired cognitive performance, measured by the MoCA, was associated with more missed HIV-care appointments after controlling for CD4 count and viral suppression status. However, the MoCA was not significantly associated with ART medication adherence. This latter finding fails to replicate the robust literature implicating NCI as a determinant of suboptimal ART adherence, particularly among PLWH who use substances [22, 23, 48]. The MoCA has demonstrated only modest sensitivity for detecting cognitive impairment among PLWH in previous research, although it has been shown to correlate with other important clinical endpoints such as clinician- and patient-assessed functional status [6, 44, 46]. Our findings extend this literature by suggesting another point of clinical utility for this brief cognitive assessment, that is, assessment for risk or suboptimal HIV-care engagement among older MSM. Our study also found that lower semantic fluency was associated with significantly fewer missed HIV-care appointments in the past year. Prior research implicated declines in verbal fluency as a characteristic feature of HIV-associated dementia. The current study contrasts the work of [49, 50], as we found a surprising inconsistency in ability to communicate effectively and maintain regular HIV-care engagement. In addition to these objective measures of cognitive performance, PAOFI self-report of cognitive complaints was also associated with more missed HIV-care appointments, suggesting that PLWH who perceive themselves to exhibit cognitive deficits may also be at increased risk of suboptimal HIV-treatment engagement.

Our analyses also suggest relationships between neurocognitive function in our sample and functional disability. Greater semantic fluency was associated with decreased functional impairment, implicating verbal fluency as a consequential cognitive domain in relation to health-related functioning in PLWH. This finding is consistent with a previous study in PLWH that identified verbal fluency as an independent correlate of successful functional aging, as well as evidence suggesting that verbal fluency is an indicator of overall executive functioning [51–53]. Additionally, self-reported cognitive issues were associated with greater functional impairment, consistent with hypotheses. However, we found that MoCA scores were not associated with functional impairment or pain after controlling for CD4 and viral suppression status. These unanticipated null effects diverge from prior findings by Fazeli et al. [6], which showed associations between the MoCA score and both patient- and clinician-assessed functional declines among older adults living with HIV [6].

We found that better performance on the TMT-B, a measure of cognitive flexibility, was associated with greater self-report pain severity. This finding stands in contrast to a substantial body of research implicating cognitive flexibility as a likely protective factor against chronic pain severity [54, 55]. For instance, meta-analytic research has shown there is impaired cognitive flexibility both among people with chronic pain [55, 56] and in people with chronic opioid exposure [57, 58]. While some prospective and experimental research studies have previously failed to show a relationship between cognitive flexibility and pain [59–61], our finding further complicates this presumed relationship in a sample of PLWH with chronic pain and SU. One potential explanation is that self-reported greater pain severity could signify a capacity to adaptably report pain differentially across contexts for functional purposes (e.g., facilitating access to opioid pain medication in response to heightened pain symptoms or altered reward signaling). However, another factor that may have contributed to this finding is that all eligible participants reported chronic pain; thus, the sample demonstrated a restricted range of pain intensity and therefore may not be representative of the relationship between pain and cognitive flexibility in PLWH more generally.

The findings of the current study should be interpreted with respect to its limitations. First, the MoCA, our measure of neurocognitive functioning, is a relatively brief screening measure and not equivalent to a comprehensive neuropsychiatric assessment. However, this brevity increases the practicality of this measurement for clinical use. Second, a cross-sectional study design precluded causal inferences or determination of temporal precedence among variables of interest. Several domains were assessed by means of self-report, which is vulnerable to confounding by biases both volitional (e.g., social desirability bias) and avolitional (e.g., recall bias). This bias may be particularly relevant with regard to the assessment of socially stigmatized and/or prohibited behaviors such as SU and domains into which participants may have had limited insight (e.g., cognitive difficulties). On the other hand, gold-standard clinician assessment of neurocognitive screening may have mitigated this concern. HIV disease control variables may have shown some reduced sensitivity due to measurement error introduced by the delay between the time of HIV lab collection (i.e., HIV RNA viral load and CD4 count) and the time of participants' study participation. Additionally, as the MoCA contains a VFT that was administered following a separate VFT administration, it is possible that a practice effect may have provided a small advantage for MoCA performance across the sample. Further, while we could not adjust for all demographic characteristics of the sample, our TMT-B scores were scaled in reference to age- and education-adjusted norms. The study could not statistically adjust for the full array of possible confounding variables. We employed multiple imputation as a means of handling missing data; while this is a robust method for minimizing possible bias in estimates and loss of power, this method relies upon the assumption that data are MCAR. Finally, smaller sample size may have impacted our ability to identify systematic relationships. Nevertheless, the central limit theorem holds that sample means tend to be approximately normally distributed in sample sizes above 30–40, and we examined the reliability of our sample mean estimates in relation to other PLWH samples.

Our findings are the first to examine these relationships among an MSM-only sample of older PWLH with chronic pain and SU and, as such, carry important implications for HIV clinical care. First, our results suggest that among MSM aging with HIV, NCI could serve as a barrier to optimal engagement in HIV care. This finding underscores a clinical need for targeted interventions to bolster attendance of HIV-care visits for PLWH who may be experiencing NCI. As impaired neurocognitive performance may engender missed visits with HIV treatment providers, this highlights the need for novel models for bolstering engagement in this population. Such support may be beyond the scope of HIV primary care, so linkage to care or healthcare navigation tailored to people aging with HIV will be important. Linkage to cognitive behavioral therapy–based interventions such as Life-Steps and medication monitoring may also improve outcomes [62]. Our results further suggest that reliance on self-report to identify NCI in older MSM living with HIV will not be sufficient due to the performance of tools such as the PAOFI. Increased neuropsychological assessment and referral from primary care may better identify aging PLWHs at risk for suboptimal treatment engagement. Addressing comorbid SUDs will also be critical for promoting optimal HIV treatment engagement. For instance, among PLWH with OUD, engagement in SUD treatment has been shown to mitigate the impact of NCI on ART adherence [63].

## 5. Conclusions

The present study suggests that NCI may contribute to suboptimal HIV treatment engagement and functional impairment among aging MSM living with HIV, chronic pain, and SU. There is a need for integrated treatments to support PLWH with NCI in clinical settings. Future research is needed to confirm and extend the findings of the present study. Longitudinal study designs utilizing objective measures of ART adherence (e.g., unannounced pill counts and digital pill technologies) are needed to better understand the causal links between NCI, SU, chronic pain, and engagement in HIV care among aging MSM living with HIV.

## Figures and Tables

**Table 1:** Participant demographic and health characteristics.

Characteristic	Mean (SD) or number (%)
	Total sample (*N* = 63)
*Demographic characteristics*	
Age (years)	57.2 (5.6)
Race	
White	34 (54.8%)
Black or African American	26 (41.9%)
American Indian/Alaskan Native	3 (4.8%)
Asian	1 (1.6%)
Native Hawaiian/Pacific Islander	1 (1.6%)
Ethnicity	
Hispanic	4 (6.6%)
Education	
HS Degree or GED	16 (27.6%)
Some college	15 (25.9%)
College degree or more	27 (46.6%)
Annual income	
Less than $10,000	15 (24.6%)
$10,000 to $20,000	30 (49.2%)
$20,000 to $40,000	13 (21.3%)
Over $80,000	13 (4.9%)

*HIV-related and general health characteristics*	
CD4 count (cells/mm^3^)	
< 200	4 (8.2%)
200–799	33 (67.3%)
≥ 800	12 (24.5%)
Viral load (mean plasma HIV RNA: copies/mL)	2473.6 (13,499.8)
Viral load (self-reported)	
Undetectable	46 (86.8%)
Detectable	7 (13.2%)
MoCA total score	23.6 (3.9)
PAOFI total score	39.7 (21.6)
Brief Pain Index composite score (0–10)	5.2 (1.6)
PEG mean score	4.9 (2.5)
Perceived physical health (0–100 VAS)	67.4 (19.6)
Functional disability score (WHO DAS 2.0)	12.7 (8.2)

*Substance use and mental health characteristics*	
HADS-depression	6.7 (4.2)
HADS-anxiety	6.6 (4.2)
Current major depressive disorder	28 (44.4%)
Current anxiety disorder	26 (41.3%)
Current posttraumatic stress disorder	10 (15.9%)
Current substance use disorder	42 (66.7%)
Current alcohol use disorder	17 (27.0%)
Current stimulant use disorder	27 (42.9%)
Current opioid use disorder	12 (19.0%)
Current cannabis use disorder	18 (28.6%)

*Note:* Percentages denote percent of valid values (excluding missing data).

Abbreviations: HADS, Hospital Anxiety and Depression Scale; PEG, pain, enjoyment, and general activity interference scale; PTSD, posttraumatic stress disorder; VAS, Visual Analog Scale; WHO DAS 2.0, World Health Organization Disability Assessment Schedule 2.0.

**Table 2:** Multivariate regressions of neurocognitive function in relation to engagement in HIV care^+^.

	*B*	SE	*β*	*t*	*p*
*ART medication adherence*					
MoCA total score	−0.896	0.451	−0.260	−1.987	0.052^∗^
Trails B time to complete	0.019	0.033	0.072	0.586	0.560
Semantic fluency	0.290	0.349	0.111	0.831	0.410
PAOFI total score	−0.034	0.084	−0.051	−0.398	0.692

*Number of missed HIV-care appointments in the past year*					
MoCA total score	−0.170	0.113	−0.201	−1.504	0.138
Trails B time to complete	−0.000	0.008	−0.009	−0.051	0.959
Semantic fluency	−0.169	0.081	−0.259	−2.073	0.043^∗^
PAOFI total score	0.041	0.019	0.262	2.147	0.036^∗^

^+^All effects control for CD4 count and HIV viral suppression status.

^∗^ *p* < 0.05.

**Table 3:** Multivariate regressions of neurocognitive function in relation to health and quality of life^+^.

	*B*	SE	*β*	*t*	*p*
*Pain (Brief Pain Index composite score)*					
MoCA total score	−0.010	0.056	−0.026	−0.184	0.855
Trails B time to complete	−0.009	0.003	−0.306	−2.352	0.022^∗^
Semantic fluency	−0.067	0.041	−0.210	−1.646	0.105
PAOFI total score	0.009	0.010	0.114	0.889	0.378

*Functional impairment (WHODAS)*					
MoCA total score	−0.087	0.280	−0.043	−0.313	0.756
Trails B time to complete	−0.035	0.019	−0.230	−1.802	0.076
Semantic fluency	−0.450	0.200	−0.283	−2.273	0.027^∗^
PAOFI total score	0.199	0.041	0.526	4.827	< 0.001^∗∗∗^

*Note:* Pain, Brief Pain Index composite score.

Abbreviations: MoCA, Montreal Cognitive Assessment; PAOFI, Patient's Assessment of Own Functioning Inventory.

^+^All effects control for CD4 count and HIV viral suppression status.

^∗^ *p* < 0.05.

^∗∗^ *p* < 0.01.

^∗∗∗^ *p* < 0.001.

**Table 4:** Comparison of neurocognitive function scores with mean scores in comparable samples of people living with HIV.

	*N*	Mean	SD	SE of difference	*t*	*p*
*MoCA*						
Present sample	62	23.6	3.9			
Milanini et al., 2016	93	24.0	2.8	0.54	0.596	0.552
Fazeli et a., 2017	100	25.2	3.0	0.54	2.886	0.004^∗∗^
Koenig et al., 2016	125	24.8	3.0	0.51	2.282	0.024^∗^
Janssen et al., 2015	95	26.6	2.3	0.49	6.014	< 0.001^∗∗∗^

*PAOFI*						
Present sample	63	39.7	21.6			
Rourke et al., 1999 CDC-A	12	50.7	23.6	6.90	1.590	0.116
CDC-B	41	55.8	28.5	4.92	3.265	0.002^∗∗^
CDC-C	47	49.0	31.3	5.04	1.837	0.069

*Note:* CDC-A to C, Center for Disease Control's Classification for stage of HIV disease; PAOFI, Patient's Assessment of Own Functioning Inventory (higher score indicates more cognitive complaints).

Abbreviations: MoCA, Montreal Cognitive Assessment.

^∗^ *p* < 0.05.

^∗∗^ *p* < 0.01.

^∗∗∗^ *p* < 0.00.

## Data Availability

The de-identified statistical dataset used to produce the current analyses is available upon request.
